# Predicting Hypocalcemia and Identifying Supplementation Needs After Total Thyroidectomy: The Role of Perioperative PTH Measurements

**DOI:** 10.3390/biomedicines14010062

**Published:** 2025-12-26

**Authors:** Angeliki Emmanouilidou, Athina Stamati, Eleni Avramidou, Philippos Tasioudis, Eleni Tziona, Charilaos Koulouris, Michael Karanikas, Kalliopi Pazaitou-Panayiotou, Nickos Michalopoulos

**Affiliations:** 1 School of Medicine, Aristotle University of Thessaloniki, 54124 Thessaloniki, Greece; 2 Department of Endocrine Surgery, Genesis Hospital, 55535 Thessaloniki, Greece; 3 European Interbalkan Medical Center, 57001 Thessaloniki, Greece

**Keywords:** hypocalcemia, total thyroidectomy, PTH, supplementation, calcium

## Abstract

**Background**: Post-thyroidectomy hypocalcemia is a common complication, yet the optimal perioperative markers for identifying high-risk patients and guiding supplementation remain debated. This study aimed to evaluate factors associated with hypocalcemia at 24 h after total thyroidectomy, identify independent predictors, and assess the reliability of early PTH measurement in determining supplementation needs. **Methods**: We conducted a single-center prospective cohort study including 200 patients undergoing total thyroidectomy at Genesis Hospital, Thessaloniki, between November 2022 and March 2025. PTH was measured preoperatively, 10 min post-resection, and at 24 and 72 h; calcium and phosphorus were measured preoperatively and postoperatively. **Results**: Independent predictors of hypocalcemia at 24 h were female sex, preoperative calcium, and PTH at 10 min. Age, pathology, incidental parathyroid excision, and extent of surgery were not significantly associated with hypocalcemia. ROC analysis showed that a preoperative calcium cutoff of 9.47 mg/dL yielded an AUC of 0.73, with 70.1% sensitivity and an NPV of 82%. PTH at 10 min with a cutoff of 24.6 pg/mL yielded an AUC of 0.66, with 70.1% sensitivity and an NPV of 79%. For supplementation needs, PTH at 10 min demonstrated excellent discrimination, with a cutoff of 16.3 pg/mL at 24 h and 14.1 pg/mL at 72 h. **Conclusions**: Preoperative calcium and PTH measured 10 min after thyroid removal are useful markers for predicting hypocalcemia after total thyroidectomy, with early PTH also accurately identifying supplementation needs.

## 1. Introduction

Thyroidectomy is a surgical procedure that involves the total or partial resection of the thyroid gland [1]. Despite thyroidectomy being a standard surgical procedure in most cases, it still carries a risk of complications [2,3,4]. Hypocalcemia, as a result of hypoparathyroidism, is amongst the most common and clinically significant. Hypoparathyroidism following thyroidectomy is categorized as either transient, when it usually resolves within a few weeks to months, or permanent, when it lasts for more than 6 months [5]. Reported incidence of transient hypocalcemia after thyroidectomy ranges significantly from 6.09% to 49%, while 3–5% of patients will develop permanent hypocalcemia [6,7,8,9].

Factors correlated with higher incidence of hypoparathyroidism arise from either the patient or the surgical procedure. Regarding surgical factors, higher prevalence of hypoparathyroidism has been reported in complicated and longer-duration (>120 min) thyroid procedures, such as total thyroidectomy with lymph node excision, when compared to a lobectomy [5,10]. Patient parameters linked with post-thyroidectomy hypoparathyroidism include genetic factors, such as DiGeorge syndrome, autoimmune disorders, such as Hashimoto’s thyroiditis, younger age (<40 years), and female sex [11,12]. These demographic variations highlight the need for tailored preoperative counseling and postoperative monitoring strategies.

Hypocalcemia after total thyroidectomy (TT) can prove to be a major complication for patients predisposing them to potentially life-threatening complications. Symptoms can range from paresthesia and tetany to more serious ones like psychiatric manifestations, seizures, and arrhythmias. Additionally, in the early postoperative period, Chvostek’s and Trousseau’s signs may indicate serious hypocalcemia [7,13].

Postoperative hypocalcemia is closely monitored and managed with routine use of oral calcium and/or vitamin D supplements [14]. While these medications successfully manage to control calcium levels of patients with postoperative hypoparathyroidism, prolonged high-dose therapy may contribute to delayed recovery of parathyroid gland function, nephrolithiasis and nephrocalcinosis [15]. Despite their widespread use, specific evidence-based guidelines are lacking when it comes to supplementing patients after a thyroidectomy, with most surgeons in favor of prophylactic rather than personalized therapy.

PTH is one of the primary hormones that control calcium blood levels and is directly linked with the function of parathyroid glands [16]. Its short half-life (~5 min) and physiologic association with calcium level balance enables it to become a promising biomarker for early prediction of hypocalcemia [17,18]. However, there is no consensus on optimal timing or interpretation of perioperative PTH measurements. Previous studies have measured PTH at various time points (10 min, 1 h, 3 h, 4 h, and postoperative day 1), but clinical protocols remain heterogeneous when it comes to predicting hypocalcemia after a thyroidectomy. Given this context, this study aimed to evaluate the predictive value of perioperative PTH levels, particularly those measured 10 min after gland resection, in relation to hypocalcemia occurring 24 h after a total thyroidectomy. Additionally, we sought to identify other factors influencing the incidence of biochemical hypocalcemia and establish potential prognostic cutoffs for hypocalcemia and supplementation needs after surgery.

## 2. Materials and Methods

A total of 200 patients who underwent total thyroidectomy for various indications during the aforementioned period were included in the study. Exclusion criteria involved incomplete preoperative or postoperative data, history of renal or liver failure, history of multiple myeloma, reoperation on the thyroid, concurrent surgery for primary hyperparathyroidism or presence of incidental adenoma during thyroid surgery, use of calcium supplements prior to surgery, vitamin D deficiency status, need for intravenous calcium and/or oral calcium, and vitamin D supplementation during hospital stay.

Data collection included patient demographics, clinical and imaging records, perioperative biochemical testing, operative findings, histopathology reports, and supplementation status. PTH, total serum calcium, and phosphorus were measured preoperatively, at 24 h (postoperative day 1) and at 72 h (postoperative day 3). Additionally, PTH values were measured 10 min after thyroid gland resection.

Total calcium and phosphorus levels were measured with a colorimetric assay whereas immunoassay was utilized for serum PTH levels determination. Normal reference ranges were 8.6–10.2 mg/dL for total serum calcium, 2.6–4.5 mg/dL for phosphorus, and 15–65 pg/mL for PTH. We defined biochemical hypocalcemia as a value of total calcium < 8.6 mg/dL regardless of the clinical symptoms.

Statistical analysis involved descriptive statistics, initially assessing the distribution of data by implementing the Shapiro–Wilk test. For normally distributed data, continuous variables were expressed using mean and standard deviation (SD), while categorical values were presented using absolute and relative frequencies. Non-normally distributed data were characterized by median and interquartile range (IQR). Univariate and multivariable binary logistic regression were performed to identify predictors of postoperative hypocalcemia. Variables that were statistically significant in univariate analysis (*p* < 0.05) and available before the outcome measurement were entered into the multivariable model. All prerequisites for running a multivariable logistic regression were checked. The logistic regression model was evaluated using the goodness-of-fit test. Chi-square tests were implemented to compare categorical variables. In order to meet test assumptions and avoid sparse data bias, pathology categories were combined into “Autoimmune Disease” for Graves’ and Hashimoto thyroiditis, “Cancer” for papillary and follicular carcinoma, and “Benign Nodular Disease” for nodular goiter and follicular adenoma cases. The Mann–Whitney U test was used for continuous, non-normally distributed variables. Receiver operating characteristic (ROC) curve analysis and Youden’s index were applied to determine the optimal cutoff values for identifying postoperative hypocalcemia and supplementation needs at 24 and 72 h after surgery. Diagnostic performance was assessed with confusion matrices, assessing sensitivity, specificity, positive predictive value (PPV), negative predictive value (NPV), and accuracy. Lastly, McNemar’s test was used for assessing paired categorical data.

The significance level was set at 0.05 for the statistical analysis which was conducted using the IBM SPSS Statistics version 29.0.2.0.

## 3. Results

### 3.1. Patient Characteristics

The characteristics and laboratory findings of the patients are shown in Table 1 and Table 2. This study included 200 patients with a mean age of 46.4 years. Out of the total number of patients, 151 (75.5%) were female and 49 (24.5%) were male. Histopathological diagnosis included papillary thyroid carcinoma in 97 (48.5%) patients, follicular adenoma in 16 (8%), Graves’ disease in 11 (5.5%), nodular goiter in 74 (37%), and single cases (0.5% each) of follicular thyroid carcinoma and Hashimoto thyroiditis. All patients underwent total thyroidectomy; 101 (50.5%) did not undergo any lymph node dissection, 76 (38%) underwent unilateral central lymph node dissection (UCLND), 16 (8%) underwent bilateral central lymph node dissection (BCLND), and only 7 (3.5%) underwent central lymph node dissection (CLND) accompanied with lateral lymph node dissection (LLND). Biochemical hypocalcemia 24 h after thyroidectomy was defined as a total serum calcium value of less than 8.6 mg/dL. Prevalence of hypocalcemia at 24 h postoperatively was 33.5%. Incidence of biochemical hypocalcemia at the same time point was 32.5%. Supplementation with calcium and vitamin D was prescribed to 58 (29%) patients at 24 h while the total number of patients receiving supplements at 72 h was 51 (25.5%).

### 3.2. Predicting Hypocalcemia 24 H After TT

To identify predictors of hypocalcemia at 24 h after surgery, we performed univariate and multivariable logistic regression. For the model to have a possible clinical value, we included only the parameters known to physicians before the patient was discharged. Thus, the parameters included in this analysis were sex, age, preoperative laboratory findings (calcium, phosphorus, PTH), PTH measured 10 min after gland resection, and the type of lymph node dissection. The results for univariate and multivariable analysis are depicted in Table 3 and Table 4, respectively.

Univariate logistic regression indicated only sex, preoperative calcium levels, and PTH 10 min after gland resection as significant predictors; thus, only these parameters were later incorporated in the multivariable logistic regression model. In the combined model, adjusting for all parameters, all variables remain statistically significant predictors of hypocalcemia at 24 h after a total thyroidectomy. Specifically, the results show that females had threefold higher odds (OR = 3.02, 95% CI 1.22–7.55, *p* = 0.02) of developing hypocalcemia compared to males. Regarding continuous variables, for every 1 mg/dL increase in the preoperative calcium levels, the odds of hypocalcemia at 24 h decrease by 91% (OR = 0.09, 95% CI 0.04–0.23, *p* < 0.001), whereas for every 1 pg/mL increase in PTH levels at 10 min after gland resection, the odds of hypocalcemia decrease by 3% (OR = 0.97, 95% CI 0.95–0.99, *p* < 0.001).

Postoperative variables, primarily obtained from the histopathology report, became available after the occurrence of hypocalcemia and were thus not included in the predictive logistic regression model to avoid look-ahead bias. Instead, continuous variables were compared between patients with and without hypocalcemia at postoperative day 1 using the Mann–Whitney U test, while categorical variables were analyzed using chi-square tests. In particular, the thyroid mass did not differ significantly between patients who developed hypocalcemia at 24 h after surgery (Mdn = 23, Q1 = 17, Q3 = 39.5) and those that did not (Mdn = 24, Q1 = 16, Q3 = 37, U = 4392, *p* = 0.87). Neither parathyroid extraction (χ^2^(1, N = 200) = 0.007, *p* = 0.934) nor pathology (χ^2^(2, N = 200) = 0.003, *p* = 0.99) differed significantly between the two groups (Supplementary Tables S1 and S2).

**Figure 1:**
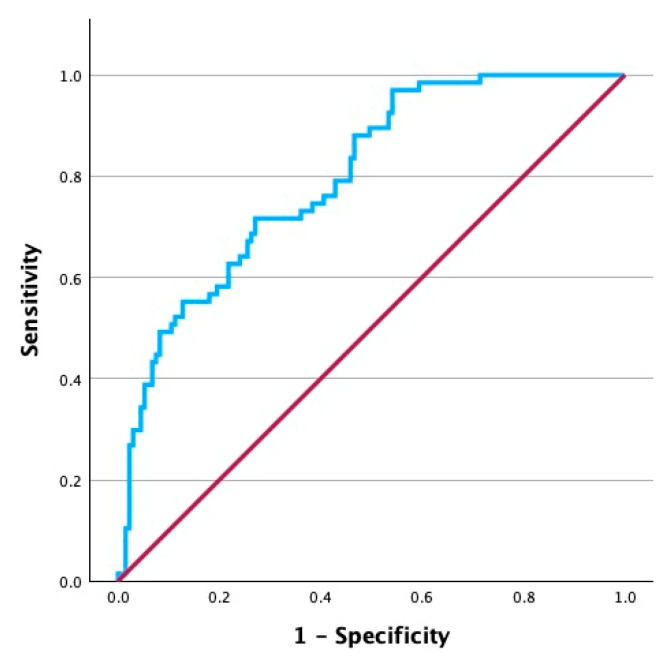
ROC curve of multivariable model for hypocalcemia at 24 h.

**Figure 2:**
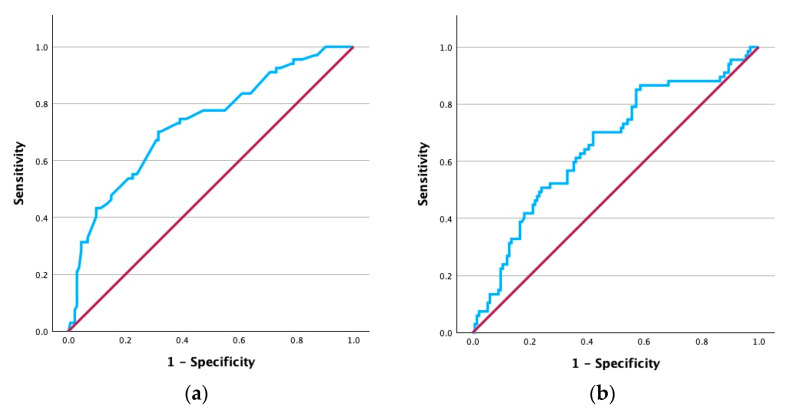
ROC curves of (**a**) preoperative calcium and (**b**) 10-minute postoperative PTH for hypocalcemia at 24 h.

ROC curve analysis was performed to assess the ability of the combined multivariable logistic regression model to discriminate between patients who did and those who did not develop hypocalcemia at 24 h after a total thyroidectomy. Figure 1 depicts the analysis which yielded an AUC of 0.80, indicating good discriminative ability. We subsequently assessed each continuous variable in the model separately to determine the optimal cutoff values that could potentially serve as a diagnostic tool. ROC curve analyses for preoperative calcium levels and PTH levels 10 min after resection are presented in Figure 2. Preoperative calcium showed good discrimination for identifying hypocalcemia 24 h after surgery, with an optimal cutoff of 9.47 mg/dL (AUC = 0.73, 95% CI 0.65–0.80, *p* < 0.001). On the other hand, PTH at 10 min demonstrated lower discrimination, with an optimal cutoff of 24.63 pg/mL (AUC = 0.66, 95% CI 0.58–0.74, *p* < 0.001).

The diagnostic performance of the selected cutoff values was further assessed using confusion matrices. A preoperative calcium cutoff of <9.47 mg/dL showed a 70.1% sensitivity, 68.4% specificity, 52.8% positive predictive value (PPV), 82% negative predictive value (NPV), and 69% overall accuracy for identifying hypocalcemia at 24 h. Similarly, PTH values at 10 min after gland resection with a cutoff of <24.63 pg/mL showed a 70.1% sensitivity, 57.8% specificity, 45.6% PPV, 79.4% NPV, and 62% accuracy.

Lastly, we wanted to explore the pattern of change, from preoperative PTH levels to 10 min after gland resection, and from that time point to 24 h after a total thyroidectomy. The results showed that among the 148 patients who experienced a drop in PTH levels between the preoperative and 10 min after gland removal measurements, 89 (60.1%) continued showing a decrease in PTH levels at 24 h after surgery. In contrast, of the 52 patients with no change or increase in PTH levels at 10 min, 46 (88.5%) exhibited a decrease in PTH levels by 24 h after surgery (Supplementary Table S3). McNemar’s test indicated no significant difference in the direction of changes between the two-time intervals (χ^2^(1, N = 200) = 1.37 *p* = 0.242).

### 3.3. Diagnostic Performance of PTH_10min_ for Guiding Post-Thyroidectomy Supplementation

Given the strong predictive association between PTH levels at 10 min and hypocalcemia, we next examined its utility as a single-marker for guiding supplementation decisions at 24 and 72 h postoperatively. PTH levels at 10 min were significantly lower in patients who required supplementation (Mdn = 9.4, Q1 = 6.3, Q3 = 13.3) compared to those who did not (Mdn = 31.5, Q1 = 19.1, Q3 = 50.2) at 24 h (U = 845, *p* < 0.001). Accordingly, a similar pattern was found when testing PTH levels between those that did (Mdn = 8.4, Q1 = 5.6, Q3 = 12.6) and those that did not receive (Mdn = 30.7, Q1 = 18.1, Q3 = 48.7) supplementation with calcium and vitamin D at 72 h (U = 653, *p* < 0.001).

**Figure 3:**
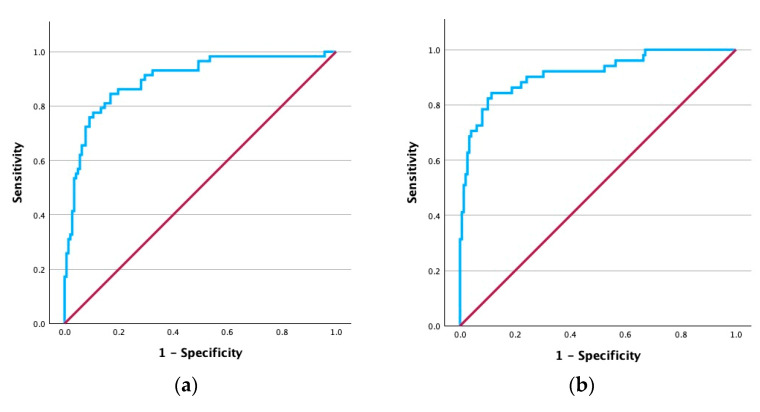
ROC curves of PTH at 10 min post-resection predicting supplementation at (**a**) 24 and (**b**) 72 h.

ROC curve analysis for PTH values was subsequently performed to explore cutoffs that could provide insight into guidance of supplementation needs. Figure 3 depicts the findings at both time points. The results indicated an excellent ability for PTH values at 10 min to discriminate between those that were given supplements and those that were not at 24 h postoperatively (AUC = 0.90, 95% CI 0.85–0.95, *p* < 0.001). Using Youden’s index, the most accurate cutoff was assigned to 16.35 pg/mL. At levels below this threshold, confusion matrix analysis indicated 84.5% sensitivity, 83.1% specificity, 67.1% PPV, 92.9% NPV, and 83.5% accuracy for identifying supplementation needs at 24 h. At 72 h, the discriminative performance was excellent (AUC = 0.91, 95% CI 0.87–0.96, *p* < 0.001) with an optimal cutoff of 14.14 pg/mL. Values below this threshold showed 84.3% sensitivity, 88.6% specificity, 71.7% PPV, 94.3% NPV, and 87.5% accuracy for identifying supplementation needs at 72 h after total thyroidectomy.

Finally, we evaluated whether there were significant changes in supplementation requirements between 24 and 72 h postoperatively. McNemar’s exact test was used to assess paired differences between these two time points. The results showed that among the 142 patients not supplemented at 24 h, 136 (95.8%) remained off supplementation at 72 h, while among the 58 supplemented at 24 h, 45 (77.6%) continued supplementation at 72 h (Supplementary Table S4). Overall, there was no statistically significant change in supplementation status between 24 and 72 h postoperatively (*p* = 0.167).

## 4. Discussion

In our prospective study, we found that biochemical hypocalcemia had a prevalence of 33.5%, consistent with the other literature reporting transient hypocalcemia ranges of 3–53% [19,20]. Utilizing total serum calcium levels for our study rather than ionized calcium was based on previous recommendations, suggesting that ionized calcium testing is more expensive and not necessarily more sensitive in guiding the management of postoperative hypoparathyroidism and consequent hypocalcemia [13]. The 24 h time point for assessing biochemical hypocalcemia was selected to coincide with routine discharge after total thyroidectomy at our institution. The ATA (American Thyroid Association) Surgical Affairs Committee states that serum calcium reaches its nadir between 24 and 72 h after thyroidectomy, a delayed decrease considering early PTH drop [13,14]. Therefore, recognizing that some cases of hypocalcemia may present later, a 72 h measurement is equally important to capture the postoperative hypocalcemia window and to dictate supplementation decisions falling within the surgeon’s responsibility.

Factors associated with hypocalcemia vary widely across the literature. In our analysis, when testing for factors known to the surgeon before the first postoperative day, we found that only sex, preoperative calcium levels, and PTH measured at 10 min post gland resection were independent predictors, as well as combined predictors, of hypocalcemia at 24 h. Women were three times more likely to develop hypocalcemia, a finding consistent with a systematic review demonstrating significantly higher incidence of transient hypocalcemia after thyroidectomy in females compared to males (OR = 2.28, 95% 1.53–3.4) [21]. In contrast to some studies indicating younger age as a risk factor for postoperative hypocalcemia, we did not find age to be significant [22].

Several studies have linked certain thyroid conditions with low calcium levels post-thyroidectomy. Histopathologic findings of malignancy and Graves’ disease have been associated with hypocalcemia, but our study found no associations among different pathologies [21,22,23]. Similarly, we did not find any association between incidental parathyroid gland extraction and hypocalcemia. In our analysis, this variable reflected whether parathyroid tissue was present on the excised specimen rather than the total number of parathyroid glands removed. Theoretically, inadvertent parathyroid gland excision could decrease PTH levels depending on the number of glands removed and PTH levels preoperatively. However, the literature findings are equivocal; some studies indicate no association with the final number of preserved parathyroids, while others report increased risk of hypocalcemia in cases of accidental removal [22,24].

All patients in our study underwent total thyroidectomy with varying extents of cervical lymph node dissection. We found that type of surgery was not significantly important in predicting hypocalcemia postoperatively. This comes into contrast with the existing literature, where higher rates of hypocalcemia are seen in more extensive and time-consuming surgeries [25]. An etiology for the lack of variation in our findings may lie in the bilateral removal of the gland in the entire cohort, eliminating the protective effect of lobectomy described in the literature [21].

Findings from our study population indicated that higher preoperative calcium levels were independently associated with a lower risk of hypocalcemia at 24 h after total thyroidectomy, with each 1 mg/dL increase reducing the odds by 91%. ROC curve analysis for preoperative calcium showed acceptable discrimination for distinguishing hypocalcemia in our data with an optimal cutoff of 9.47 mg/dL. Above this threshold, patients were highly unlikely to develop hypocalcemia postoperatively. Additional studies complement our findings, demonstrating that depleted preoperative calcium levels are significantly associated with hypocalcemia after thyroidectomy [17,23,25,26]. Despite several studies included in the systematic review by Edafe et al. [21] displaying lower preoperative calcium levels in patients who later went on to develop transient hypocalcemia, the meta-analysis (n = 2493) that followed indicated no statistically significant difference between the two patient groups, although they warned of high heterogeneity (*p* < 0.001, I^2^ = 87%). Similarly, a study by Essa et al. [27], despite finding no significant difference in mean preoperative calcium levels between groups, mentions preoperative calcium as a significant predictor of postoperative hypocalcemia. All in all, we believe that preoperative calcium may be useful in identifying low risk patients.

For our analysis, we measured PTH 10 min after gland resection and found that it was an independent predictor of hypocalcemia at 24 h, with each 1 pg/mL increase reducing the odds by 3%. At a cutoff of 24.6 pg/mL, this measurement achieved 70% sensitivity and 58% specificity, and patients with values above this threshold were unlikely to develop hypocalcemia, reflected by a negative predictive value of 79%. Similar findings have been reported in other studies. A prospective analysis of 100 patients showed that a 10 min PTH cutoff of 23 pg/mL demonstrated the best compromise between specificity and sensitivity for predicting hypocalcemia [27]. Mattoo et al. [19], studying 111 patients, found that a 20 min PTH threshold of 40.36 pg/L offered 81.7% sensitivity and 51% specificity for clinically significant hypocalcemia. PTH measurements gathered later were generally more reliable. Inversini et al. [28] reported that a 3 to 6 h cutoff of 10 pg/mL was the most accurate and specific for predicting hypocalcemia, while Thakur et al. [24] demonstrated that 4 and 12 h thresholds of 13.7 pg/mL and 12.4 pg/mL, respectively, had good predictive ability for hypocalcemia at the same time points, with AUCs of 0.78 and 0.89. A smaller prospective series of 49 patients reported that a 4 h cutoff of 17.52 pg/mL provided 100% sensitivity and NPV for postoperative hypocalcemia [29]. Essa et al. showed that a 48 h cutoff of 10 pg/mL offered excellent diagnostic performance, with 100% sensitivity, 100% specificity, and an AUC of 1. These results indicate that early PTH measurements, such as those at 10 or 20 min, can warn of hypocalcemia risk; however, later assessments often demonstrate stronger diagnostic performance.

In our cohort, PTH measured at 10 min after resection demonstrated excellent accuracy in identifying patients who required supplementation, both at 24 and 72 h. Patients who eventually required calcium and vitamin D had significantly lower early PTH values, and ROC curve analysis confirmed excellent discrimination (AUC ≥ 0.90). At 24 h, a cutoff of 16.3 pg/mL correctly identified supplementation needs with 84.5% sensitivity and 83.1% specificity, while at 72 h, a threshold of 14.1 pg/mL provided 84.3% sensitivity and 88.6% specificity. In both cases, negative predictive values exceeded 90%, indicating that patients with PTH values above these cutoffs almost never required calcium and vitamin D replacement. This supports the use of selective supplementation guided by biochemical PTH levels which in turn leads to reducing overtreatment, avoiding complications related to unnecessary therapy, and potentially enabling earlier discharge [30,31]. While some advocate for routine prophylactic supplementation in all patients following total thyroidectomy, others argue for a personalized approach based on early postoperative PTH values [13,32,33,34]. A systematic review highlighted considerable variation in timing, assay type, and cutoff values used for perioperative PTH measurement worldwide and recommended that individual centers should establish PTH-based protocols for guiding calcium supplementation [21]. Collectively, our findings reinforce the role of early PTH measurement as a practical and accurate tool for directing supplementation, improving patient safety, and optimizing postoperative care.

### Limitations

Our study has certain limitations that should be acknowledged. We decided to measure and use for our analyses the total serum calcium rather than the ionized form, which is the physiologically active variant in the body. The reason for our choice was that total serum calcium is more routinely used in clinical practice which in turn is attributed to its availability, cost-effectiveness and common presence in research protocols. Another limitation of our study was that certain biochemical measurements at the postoperative period did not precisely align with our 72 h time point. This is because weekends and/or holidays coincided with our postoperative 3-day window, which led us to include very few postoperative day 4 and 5 values in our analysis. We mention this because it may have caused variability in laboratory assessment timing and supplementation adjustment. Finally, logistic regression was not used to assess if PTH is a predictor of supplementation at 24 and 72 h postoperatively. The reason for this was the fact that certain assumptions for the regression analysis were violated and would call for transformation of PTH values, further compromising clinical interpretability. Instead, we opted for the Mann–Whitney U test to preserve the relevance of the original PTH values.

## 5. Conclusions

Preoperative calcium and PTH at 10 min after gland resection are reliable predictors of hypocalcemia, with perioperative PTH showing strong potential for guiding supplementation decisions after total thyroidectomy. Further multicenter prospective studies are warranted to validate these findings and refine safe, individualized supplementation and discharge protocols.

## Figures and Tables

**Table 1:** Descriptive analysis of demographic and clinicopathologic characteristics in study population.

Characteristic	Value
N	200
Age (years) ^a^	46.4 ± 14.39
Sex ^c^	
Male	49 (24.5%)
Female	151 (75.5%)
Pathology ^c^	
Papillary cancer	97 (48.5%)
Follicular adenoma	16 (8%)
Graves’ disease	11 (5.5%)
Nodular goiter	74 (37%)
Follicular cancer	1 (0.5%)
Thyroiditis Hashimoto	1 (0.5%)
Thyroid mass (g) ^b^	24 (16–37.75)
Parathyroid extracted ^c^	
Yes	53 (26.5%)
No	147 (73.5%)
Lymph node dissection ^c^	
No	101 (50.5%)
UCLND	76 (38%)
BCLND	16 (8%)
CLND and LLND	7 (3.5%)
Supplementation 24 h ^c^	
Yes	58 (29%)
No	142 (71%)
Supplementation 72 h ^c^	
Yes	51 (25.5%)
No	149 (74.4%)
Hypocalcemia 24 h ^c^	
Yes	67 (33.5%)
No	133 (66.5%)

^a^ Mean ± standard deviation. ^b^ Median (interquartile range). ^c^ Number (percentage).

**Table 2:** Descriptive analysis of laboratory findings in study population.

Laboratory Parameter	Value	Range
Preoperative calcium ^b^	9.51 (9.22–9.8)	8.3–11.5
Preoperative phosphorus ^b^	3.5 (3.1–3.6)	2.1–4.9
Preoperative PTH ^b^	40.1 (32.14–49.61)	14.03–164.1
PTH 10 min after gland resection ^b^	22.93 (12.58–39.44)	2.66–116.1
Postoperative 24 h calcium ^b^	8.7 (8.42–9)	7.6–10
Postoperative 24 h phosphorus ^a^	4.14 ± 0.71	2.3–6.2
Postoperative 24 h PTH ^b^	21.82 (11.36–31.83)	1.75–101.3
Postoperative 72 h calcium ^a^	9.13 ± 0.58	6.62–11
Postoperative 72 h phosphorus ^b^	3.94 (3.58–4.5)	2.6–7.06
Postoperative 72 h PTH ^b^	25.62 (13.83–37.19)	1.2–150.4

^a^ Mean ± standard deviation. ^b^ Median (interquartile range).

**Table 3:** Univariate logistic regression analysis for possible predictors of hypocalcemia at 24 h.

Variables	Odds Ratio	95% Confidence Interval	*p*-Value
Sex ^a^	3.29	1.44–7.5	0.005
Age (years)	0.98	0.96–1	0.08
Preoperative calcium	0.13	0.06–0.29	<0.001
Preoperative phosphorus	1.17	0.57–2.42	0.662
Preoperative PTH	1.01	0.99–1.02	0.39
PTH 10 min after gland resection	0.98	0.96–0.99	0.003
Lymph node dissection ^b^			
UCLND	1.13	0.6–2.12	0.69
BCLND	0.69	0.2–2.29	0.54
CLND and LLND	1.54	0.38–7.3	0.58

^a^ Reference category is male. ^b^ Reference category is no dissection.

**Table 4:** Multivariable logistic regression analysis for predicting hypocalcemia at 24 h.

Variables	Odds Ratio	95% Confidence Interval	*p*-Value
Sex ^a^	3.02	1.22–7.5	0.02
Preoperative calcium	0.09	0.04–0.23	<0.001
PTH 10 min after gland resection	0.97	0.95–0.99	<0.001

^a^ Reference category is male.

## Data Availability

Data is contained within the article or Supplementary Material.

## Supplementary Materials

The following supporting information can be downloaded at: https://www.mdpi.com/article/10.3390/biomedicines14010062/s1, Table S1: Association between parathyroid gland removal and postoperative hypocalcemia Table S2: Association between pathology categories and postoperative hypocalcemia, Table S3: Association between PTH rate of change from preoperative to postoperative measurements, Table S4: Association of supplementation patterns at 24 and 72 h after thyroidectomy.

## Abbreviations

The following abbreviations are used in this manuscript:

TT	Total Thyroidectomy
PTH	Parathyroid Hormone
SD	Standard Deviation
IQR	Interquartile Range
ROC	Receiver Operating Characteristic
PPV	Positive Predictive Value
NPV	Negative Predictive Value
UCLND	Unilateral Central Lymph Node Dissection
BCLND	Bilateral Central Lymph Node Dissection
OR	Odds Ratio
CI	Confidence Interval
Mdn	Median
Q1	First Quartile
Q3	Third Quartile
U	Mann–Whitney U test
N	Number
AUC	Area Under Curve
ATA	American Thyroid Association
