# Supplementary material for: Predicting Hypocalcemia and Identifying Supplementation Needs After Total Thyroidectomy: The Role of Perioperative PTH Measurements
Source: Biomedicines. 2025 Dec 26;14(1):62. doi: 10.3390/biomedicines14010062 (PMC12837889; doi:10.3390/biomedicines14010062)
Supplement: Supplementary file 1 [file biomedicines-14-00062-s001.zip › Supplementary T1.pdf]

**Supplementary Table S1.** Association between parathyroid gland removal and postoperative hypocalcemia

| Hypocalcemia at 24 hours | Parathyroid on specimen | | |
|---|---|---|---|
| | No | Yes | Total |
| No | 98 | 35 | 133 |
| Yes | 49 | 18 | 67 |
| Total | 147 | 53 | 200 |
