# Supplementary material for: Predicting Hypocalcemia and Identifying Supplementation Needs After Total Thyroidectomy: The Role of Perioperative PTH Measurements
Source: Biomedicines. 2025 Dec 26;14(1):62. doi: 10.3390/biomedicines14010062 (PMC12837889; doi:10.3390/biomedicines14010062)
Supplement: Supplementary file 1 [file biomedicines-14-00062-s001.zip › Supplementary T2.pdf]

Supplementary Table S2. Association between pathology categories and postoperative hypocalcemia

Hypocalcemia at 24 hours	Pathology			
	Benign nodular disease	Autoimmune disease	Cancer	Total
No	60	8	65	133
Yes	30	4	33	67
Total	90	12	98	200
