# Supplementary material for: Predicting Hypocalcemia and Identifying Supplementation Needs After Total Thyroidectomy: The Role of Perioperative PTH Measurements
Source: Biomedicines. 2025 Dec 26;14(1):62. doi: 10.3390/biomedicines14010062 (PMC12837889; doi:10.3390/biomedicines14010062)
Supplement: Supplementary file 1 [file biomedicines-14-00062-s001.zip › Supplementary T3.pdf]

Supplementary Table S3. Association between PTH rate of change from preoperative to postoperative measurements

PTH change from pre-op to 10min post resection	PTH change from 10 min to 24 hours		
	Decrease	Increase	Total
Decrease	89	59	148
Increase	46	6	52
Total	135	65	200
