# Supplementary material for: Predicting Hypocalcemia and Identifying Supplementation Needs After Total Thyroidectomy: The Role of Perioperative PTH Measurements
Source: Biomedicines. 2025 Dec 26;14(1):62. doi: 10.3390/biomedicines14010062 (PMC12837889; doi:10.3390/biomedicines14010062)
Supplement: Supplementary file 1 [file biomedicines-14-00062-s001.zip › Supplementary T4.pdf]

Supplementary Table S4. Association of supplementation patterns at 24 and 72 hours after thyroidectomy

Supplementation at 24 hours	Supplementation at 72 hours		
	No	Yes	Total
No	136	6	142
Yes	13	45	58
Total	149	51	200
